# *Enterococcus faecium* Bacteriophage vB_EfaH_163, a New Member of the *Herelleviridae* Family, Reduces the Mortality Associated with an *E. faecium* vanR Clinical Isolate in a *Galleria mellonella* Animal Model

**DOI:** 10.3390/v15010179

**Published:** 2023-01-07

**Authors:** Inés Pradal, Angel Casado, Beatriz del Rio, Carlos Rodriguez-Lucas, Maria Fernandez, Miguel A. Alvarez, Victor Ladero

**Affiliations:** 1 Department of Technology and Biotechnology of Dairy Products, Dairy Research Institute, IPLA-CSIC, 33300 Villaviciosa, Spain; 2 Instituto de Investigación Sanitaria del Principado de Asturias (ISPA), 33011 Oviedo, Spain; 3 Microbiology Laboratory, Hospital el Bierzo, 24404 Ponferrada, Spain; 4 Microbiology Laboratory, Hospital Universitario de Cabueñes, 33394 Gijón, Spain

**Keywords:** *Enterococcus faecium*, phage therapy, vancomycin-resistance, phage genomics, *Galleria mellonella*

## Abstract

The rise of antimicrobial resistant (AMR) bacteria is a major health concern, especially with regard to members of the ESKAPE group, to which vancomycin-resistant (VRE) *Enterococcus faecium* belongs. Phage therapy has emerged as a novel alternative for the treatment of AMR infections. This, however, relies on the isolation and characterisation of a large collection of phages. This work describes the exploration of human faeces as a source of new *E. faecium*-infecting phages. Phage vB_EfaH_163 was isolated and characterised at the microbiological, genomic, and functional levels. vB_EfaH_163 phage, a new member of *Herelleviridae*, subfamily *Brockvirinae*, has a dsDNA genome of 150,836 bp that does not harbour any virulence factors or antibiotic resistance genes. It infects a wide range of *E. faecium* strains of different origins, including VRE strains. Interestingly, it can also infect *Enterococcus faecalis* strains, even some that are linezolid-resistant. Its capacity to control the growth of a clinical VRE isolate was shown in broth culture and in a *Galleria mellonella* animal model. The discovery and characterisation of vB_EfaH_163 increases the number of phages that might be used therapeutically against AMR bacteria.

## 1. Introduction

Since the mid-20th century, antibiotics have provided a means of successfully treating bacterial infections. However, antibiotic-resistant bacteria have been isolated since their early use. Today, antimicrobial resistant (AMR) bacteria are a huge problem; in Europe alone they are responsible for 133,000 deaths in 2019 [1] and cost health services some €1500 million every year [2]. In this worrisome scenario, the World Health Organization (WHO) listed the most threatening AMR bacteria as *Enterococcus faecium*, *Staphylococcus aureus*, *Klebsiella pneumoniae*, *Acinetobacter baumannii*, *Pseudomonas aeruginosa*, and *Enterobacter* spp., together known as the ESKAPE group [3,4]. Given their impact in terms of nosocomial infections and global health, the WHO encouraged the scientific community to search for new ways to combat these pathogens [4].

*Enterococcus faecium* is a coccus-shaped, Gram-positive bacterium that is tolerant to harsh conditions and has a versatile metabolism, allowing it to adapt to a wide variety of environments [4,5]. Together with other species of the genus *Enterococcus*, such as *Enterococcus faecalis*, it is considered a commensal bacterium of the human gastrointestinal system [4,5,6]. However, it can behave as an opportunistic pathogen, causing root canal infections [7], persistent endodontic infections [7], urinary infections [8], pneumonia [9], endocarditis [5], and bacteraemia [10]. Indeed, *E. faecium* is currently behind 90% of enterococcal infections, mainly nosocomial [5]. Unfortunately, the treatment of *E. faecium* infections is becoming more difficult due to the increasing number of AMR strains [5].

*Enterococcus* spp. are intrinsically resistant to cephalosporins, to moderate levels of aminoglycosides, and to streptogramins [6,11]; ampicillin–aminoglycoside combination therapy was therefore used against them for decades [12]. However, *E. faecium* strains resistant to ampicillin are becoming ever more common [5], and over recent years vancomycin has become the treatment of choice. Worryingly, a sharp increase in vancomycin-resistant (VRE) isolates has now been noted [6,11,13]. This has led to the use of last resort antibiotics such as daptomycin (a lipopeptide antibiotic), linezolid (an oxazolidinone), and their combination with the above antibiotics [6,11,12]. However, not all strains are daptomycin-susceptible, and recently a linezolid-resistance mechanism has been described in *E. faecium* and resistant strains have been isolated [6,11,12,14]. Other antibiotics, such as fosfomycin or ertapenem, and new combinations with the above last resort antibiotics (for example daptomycin with ceftaroline ertapenem, linezolid with doxycycline, gentamycin, or tigecycline) are now being studied in vitro (no efficacy in vivo has yet been showed) [12]. Thus, serious *E. faecium* infections have a bad prognosis [5], and alternative treatments are needed. A similar scenario has been described for *E. faecalis* too, with an increase in the number of resistant isolates detected, including a strong increase in linezolid-resistant isolates [14].

Novel ways of combating ESKAPE microorganisms are now under study, including antimicrobial peptides, photodynamic light therapy, silver nanoparticles, and bacteriophage therapy [2,3]. Phage therapy would seem very promising [15]. It was in fact used in the last century before antibiotics were discovered [16], but given the antibiotic revolution, fell into neglect [3,17]. Phages are viruses that infect and kill bacteria [17]. Their use as therapeutic tools has several advantages: they are strongly host-specific and harmless to humans (only the targeted bacteria are disrupted); they resist adverse conditions; they are easy to isolate; they are found wherever their hosts exist; and they are cheaply and relatively easily raised in large numbers [18,19]. In addition, phage populations are automatically controlled. They are strict parasites, so the population increases when the host is present, and decreases when the host is absent [17]. However, before a phage can be proposed as a therapeutic candidate, it must be ensured that it carries no virulence or antibiotic resistance genes, integrases, or repressors of the lytic cycle (temperate phages need to be ruled out). Neither can they be transducer phages (to avoid gene transfer between hosts), and the more strains they infect, the better [3,19].

Different studies have reported the use of *Enterococcus* phages in different settings, including biofilms [4,20], root canal infections [21], a fibrin clot model [22], food production [23,24], and a mouse model [25,26]. There is even a case report of the use of phage therapy in a human patient with chronic prostatitis [27]. In the present work, a new bacteriophage, named here as *Enterococcus faecium* bacteriophage vB_EfaH_163, was isolated from human faeces. Its morphology, host range, one-step growth curve, and genome sequence were determined, and its ability to control VRE *E. faecium* in vitro and in vivo were examined. The results highlight the potential use of this phage for controlling VRE *E. faecium* and thus improving the treatment of infections caused by this bacterium.

## 2. Materials and Methods

Ethical approval for this study was obtained from the Bioethics Committee of the CSIC (Consejo Superior de Investigaciones Científicas) and from the Regional Ethics Committee for Clinical Research (Servicio de Salud del Principado de Asturias n° 353/19) in compliance with the Declaration of Helsinki. All experiments were carried out in accordance with approved guidelines and regulations.

### 2.1. Sample, Strains and Culture Conditions

A faecal sample was obtained from a volunteer and stored until being processed in a GutAlive device (Microviable Therapeutics, Gijón, Spain) at the IPLA facilities.

Five *E. faecium* strains, LMGY1, LMGY-10, AM, HF52, and LGM11397 of different origins (Table 1) were used for phage screening. In addition, a total of 77 *E. faecium* and 11 *E. faecalis* strains were challenged by the double-layer agar spot test to determine the host range of the vB_EfaH_163 phage (Table 1).

All bacteria were grown in M17 medium (Formedium, Swaffham, UK) supplemented with glucose (0.5% p/v) (GM17), plus 10 mM CaNO_3_ and 10 mM Mg_2_SO_4_ for the screening experiment (CaMg-GM17). Broth cultures and plates were incubated at 37 °C with no agitation.

### 2.2. Phage Titre Determination

Phage titres were determined by the double-layer agar technique using 10-fold serial dilutions. A 10 µL spot of serial phage dilution was placed on a 2% agar plate. After the drops dried, a second agar layer (5 mL of GM17 with 1.2% agar) mixed with 300 µL of an overnight culture of corresponding host strain was added. Plaques were counted after overnight incubation at 37 °C.

### 2.3. Phage Isolation and Propagation

Phage vB_EfaH_163 was isolated from the human faecal sample by enrichment culture and following the spot method in double-layer agar plates [23]. Enrichment cultures were inoculated with 100 µL of overnight cultures of the selected *E. faecium* strains and 100 µL of faecal sample processed following a previously described procedure [31]—except that after gradient centrifugation the supernatant was not discarded but used as a source of bacteriophages in enrichment cultures that were incubated overnight at 37 °C. The tubes were then centrifuged at 2000× *g* for 15 min (5910R Eppendorf benchtop centrifuge) and 100 µL of the supernatant added to 100 µL of overnight cultures of the same strain. Two such enrichment rounds were performed. Finally, 10 µL of the supernatant were spotted onto double-layered agar CaMg-GM17 plates covered with the second layer inoculated with the aforementioned *E. faecium* strains and incubated for 24 h at 37 °C. When an inhibition halo was observed, the source supernatant was streaked to obtain single plaques. For bacteriophage purification, a single plaque was picked up with a sterile tip, inoculated into 50 mL of CaMg-GM17 broth inoculated with the host strain, and incubated at 30 °C for 6 h. The culture was then centrifuged at 4000 rpm in a 5910R Eppendorf benchtop centrifuge, the phage titre determined, and the supernatant stored at 4 °C.

To obtain larger and more concentrated phage stocks, infections were performed in larger volumes of GM17 media (from 100 mL up to 600 mL) inoculated at 1% with an overnight culture of the host strain. After incubation at 25 °C for 1 h, phage vB_EfaH_163 was added at an MOI of 10 and incubated for 18 h at the same temperature. Finally, the culture was centrifuged for 15 min at 4000 rpm as above, the phage titre determined, and the supernatant stored at 4 °C.

### 2.4. Electron Microscopy

Phage vB_EfaH_163 was concentrated using the PEG/NaCl method [32]. Electron microscopy images were obtained as previously described [33]: phage particles were stained with 2% uranyl acetate solution, and electron micrographs produced using a CCD Gatan Erlangshen ES 1000 W camera coupled to a JEOL JEM 1011 transmission electron microscope (JEOL USA, Inc., Peabody, MA, USA) operating at 100 kV (performed at the Electron Microscopy Service of the Biotechnology National Centre [CNB-CSIC], Madrid, Spain).

### 2.5. One-Step Growth Curve

To construct a one step growth curve, a 1% inoculum of an overnight culture of the host strain *E. faecium* LMGY1 was added to GM17 medium. After 1 h of incubation at 37 °C, phage vB_EfaH_163 was added at an MOI of 0.1 and a sample was examined for its phage titre. After 5 min of adsorption at 37 °C, cells were harvested by centrifugation (4000 rpm for 10 min in a 5910R Eppendorf benchtop centrifuge), the phage titre of the supernatant determined, and the cells resuspended in new medium and incubated at 37 °C. At regular intervals of 15 min, samples were collected for phage titration. This experiment was performed in triplicate. The burst time and latent period were calculated from the one step growth curve produced.

### 2.6. Phage Genome Sequencing and Analysis

Phage DNA was isolated from a concentrated phage suspension obtained following the PEG/NaCl method [32] as previously described [23]. A genomic library of 0.5 kbp was constructed and subjected to 150 paired-end sequencing (providing approximately 800-fold coverage) using an Illumina HiSeq 1000 System sequencer at GATC services (Eurofins Genomics, Ebersberg, Germany). Quality filtered reads without trimming were assembled using SPADES software [34]. Annotation was performed using the RAST, (with RASTtk pipeline) [35] and PATRIC [36] servers, and improved with BLAST analysis results [37]. Automatic and manual annotation was checked for the presence of virulence factors, including toxin-enconding genes, as well as antibiotic resistance genes. Moreover, PhageLeads [38] and the Resistance Gene Identifier from the Comprehensive Antibiotic Resistance Database (CARD) [39] were used to check the presence of temperate lifestyle genes, antimicrobial resistance, and virulence genes. The genome sequence was deposited in the European Nucleotide Archive (ENA) under accession number CAJDKA010000002.1. The packaging mechanism was determined in silico using the PhagTerm tool [40] at http://galaxy.pasteur.fr (accessed on 10 February 2022). To confirm the circularity of the genome, as well as the sequence of the redundancies, a PCR amplification using primers 163f (5’-GCCCAGAATACATCCGACAAG-3´) and 163r (5´-CCAAGCCCACAAGGAACCTCC-3,) located at both contigs ends and further sequencing of the obtained amplicon was performed.

### 2.7. Phylogenetic Analysis

The amino acidic sequence of the major capsid protein of vB_EfaH_163 was aligned with those of other *E. faecium*-infecting phages for which complete genomes are available (Table 2). This was achieved using the unweighted pair group method with arithmetic means (UPGMA) and employing MAFFT v.7 software (https://mafft.cbrc.jp/alignment/server/ [41] accessed on 14 November 2022). The phylogenetic tree produced was visualized using the iTOL web server (https://itol.embl.de/ [42] accessed on 14 November 2022).

### 2.8. Technological Characterisation

The effect of different incubation temperatures on plaque formation was determined by calculating phage titres of the same phage suspension on plates incubated at different temperatures (22, 27, 32, 37, or 42 °C). The thermal stability of phage vB_EfaH_163 was tested by placing 100 µL of its suspension at different temperatures (room temperature [control], 40, 45, 50, 55, 60, 70, 80, 90 °C) for 15 min and determining phage titres. Finally, the pH stability of the phage was tested by mixing 10 µL of its suspension in 900 µL of PBS at different pHs (2–9). Phage titres were determined after 15 min of incubation. All experiments were performed in triplicate.

### 2.9. Functional Characterisation

#### 2.9.1. Biocontrol of the *E. faecium* VR-13 vanR Clinical Isolate by vB_EfaH_163 Infection in Broth

*E. faecium* VR-13 was chosen as a VRE clinical strain to test the potential of phage vB_EfaH_163 as a biocontrol tool. To evaluate its effectiveness in broth, 10 mL of GM17 medium were inoculated with an overnight culture of *E. faecium* VR-13 to obtain an initial cell concentration of (10^4^ CFU/mL). After 1 h of incubation at 37 °C, 100 µL of vB_EfaH_163 phage were added at different MOIs (0.1, 1, and 10). Total viable cells were then measured at 5 h, 7 h, and 24h. Three biological and two technical replicates for each condition were performed.

#### 2.9.2. In Vivo Effectiveness of Phage Treatment in the *Galleria mellonella* Model

vB_EfaH_163 was evaluated for its capacity to eliminate *E. faecium* infection in vivo using a *G. mellonella* (wax moth larvae) model. TruLarv larvae obtained from BioSystems Technology (Exeter, UK) were stored at 15 °C until the day of the experiment. Larvae were considered dead only when there was no movement after stimulation or when melanisation was seen, and considered alive when there was movement and no melanisation [50,51].

To test the lethality of *E. faecium* VR-13, an overnight culture of this bacterial strain was centrifuged for 10 min at 3500 rpm and the pellet resuspended in the same volume of PBS. To determine what concentration of larvae to use in experiments, 10 µL of the above suspension were injected into the right pro-leg at test concentrations of 10^5^–10^7^ CFU/larva, as previously described [52]. A group of larvae was injected with PBS as a control (see Section 3.6 for details). After inoculation, the larvae were kept in Petri dishes at 37 °C, and the number of deaths monitored for five days.

To test the effectiveness of the vB_EfaH_163 phage against *E. faecium* VR-13, an overnight culture of this bacterial strain was centrifuged for 10 min at 3500 rpm and the pellet resuspended in the same volume of PBS. The bacterium was injected into the second-last right proleg at 10^5^ CFU/larva. The control group was injected with PBS. After 1 h at 37 °C, 10 µL of the phage suspension (concentrated by the PEG-NaCl method and resuspended in sterile dH_2_O) was injected into the second last left pro-leg at an MOI of 0.1. At the same moment, control larvae were injected with sterile dH_2_O. After inoculation, all larvae were kept in Petri dishes at 37 °C, and the number of deaths monitored for five days.

### 2.10. Statistical Analysis

Means (± standard deviations) were calculated from at least three independent results and compared using the Student *t*-test. Significance was set at *p* < 0.05.

The survival curve for, and analysis of, the in vivo experiments in the *G. mellonella* model were performed using GraphPad Prism 6 following the method of Kaplan and Meier, calculating the 95% confidence interval for fractional survival at any given time. Survival curves were compared using the log-rank test. At least 10 larvae were used for each condition. Significance was set at *p* < 0.05.

## 3. Results

### 3.1. Phage vB_EfaH_163 Isolation

*E. faecium* bacteriophage vB_EfaH_163 was isolated from a human faecal sample processed following a previously described method to separate different components of the faecal stool and then isolate the faecal microbiota [31]. The method was adapted to recover the supernatant after the gradient separation of faecal components. An amount of 100 µL of this supernatant were added to CaMg-GM17 cultures, individually inoculated with one of the five potential host strains indicated in the Material and Methods section. After two rounds of enrichment culture, a growth inhibition halo was observed on *E. faecium* LMGY1 plates. The culture supernatant was then streaked to obtain isolated plaques and confirm that the inhibition halo was caused by a phage. Since only one type of plaque was observed it was assumed that only one phage was present in the sample. One of the plaques was used to infect *E. faecium* LMGY1 in broth to obtain a high titre bacteriophage stock for further characterisation. The efficiency of infection with the obtained stock was then determined in CaMg-GM17, Ca-GM17, Mg-GM17, and GM17 media. No significant differences were seen between the different media (data not shown), so the following experiments were all performed in GM17 medium.

### 3.2. Microbiological Characterisation of Phage vB_EfaH_163

To determine the host range of phage vB_EfaH_163, 77 *E. faecium* strains of different origin (Table 1) were subjected to the spot test. The phage infected about 50% of them—39 in total, of which 20 had been isolated from food, three from human faecal samples, and 16 of which were vancomycin-resistant clinical isolates (Table 1). In addition, since some *E. faecium* phages were recently shown capable of infecting some *E. faecalis* strains [4], 11 such strains of different origin were tested as possible host strains (Table 1). Two were susceptible to infection by the phage, notably one that was a linezolid-resistant clinical isolate.

**Figure 1:**
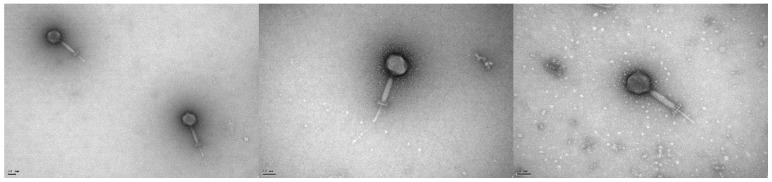
Electron photomicrographs of phage vB_EfmH_163. The scale bar represents 50 nm.

Electron photomicrographs revealed phage vB_EfaH_163 to have typical myovirus morphology (Figure 1). It had an isometric head (around 81 ± 5 nm), a contracted tail sheath length of 106 ± 7 nm finished with a decorated baseplate, and a tail tube length of 74 ± 10 nm that accounted for a total tail length of 180 ± 17 nm (Figure 1). The release of DNA from some particles can be observed (Figure 1).

**Figure 2:**
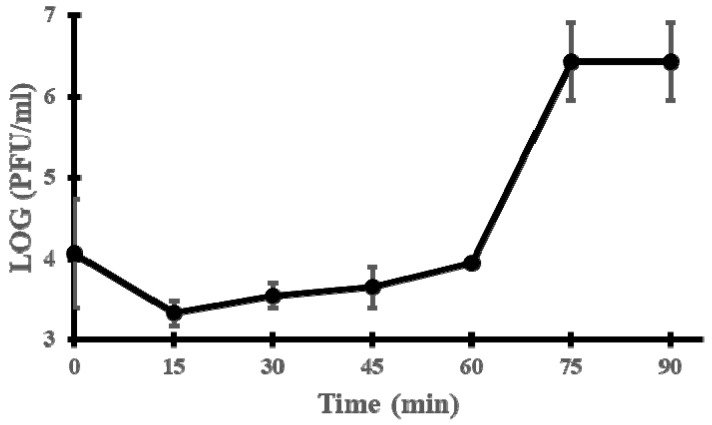
One-step growth curve of phage vB_EfmH_163. Log PFU/mL was measured every 15 min. Means and standard deviations of three independent replicates are shown.

To better characterise the phage cycle, a one-step growth curve was constructed. The latent period was estimated to last around 60 min; the burst size was about 155 PFU per infected cell (Figure 2).

### 3.3. The vB_EfmH_163 Genome: Characterisation and Phylogenetic Analysis

Assembly of the corresponding Illumina reads revealed a single contig of 150,836 bp, representing the phage genome. The GC content was 37% similar to that of the bacterial host *E. faecium* (37.8%).

**Figure 3:**
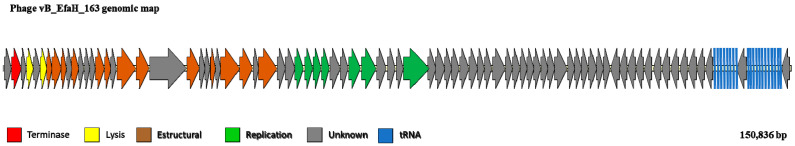
Representation of the 150,836 bp-long genome of phage vB_EfmH_163. Open reading frames are indicated by arrows pointing in the transcription direction, and coloured according to the functional module. Red: packaging; yellow: lysis genes; brown: structural genes; green: replication; grey: unknown function. *tRNA* genes are represented by thick blue lines.

Annotation, combining the RAST, PATRIC, and BLAST results, revealed the existence of 186 *orfs* and 21 *tRNAs*. Most of these *orfs* (81%) showed no similarity to genes of a known function and were consequently annotated as coding for hypothetical phage proteins. Genes for which a putative function could be assigned were mainly related to basic phage functions such as packaging (large terminase unit), lysis (endolysins with a muramidase domain), structural proteins (portal, major head, tail sheaths, tail measure, base plate), or replication (helicase, primase, DNA polymerase) (Supplementary Table S1; Figure 3). These genes are functionally grouped into modules that are all transcribed in the same direction (Figure 3), except for a small group of *orfs* of unknown function, as well as the *tRNA* genes, which are divergently transcribed (Figure 3). No genes involved in the establishment of a lysogenic cycle were found, suggesting that vB_EfaH_163 is a lytic bacteriophage. No genes related to pathogenicity, virulence (including toxin-encoding), or AMR were detected either, supporting the idea that vB_EfaH_163 could be therapeutically used without danger. The vB_EfaH_163 DNA packaging mechanism was determined in silico using the Phageterm tool, suggesting a mechanism of long direct terminal repeats, similar to that seen in phage T5 [53]. The size of the terminal repeat was estimated at 2105 bp between positions 104,991 and 107,095 of the reported genome sequence (Accession number CAJDKA010000002.1). As most of the phages with this type of packaging have a circular genome, but the obtained genome was a linear contig, a PCR amplification using primers located at both contig ends was performed. The sequencing of the resulted amplicon allowed confirmation that the genome is circular and to correct the genome sequence.

BLASTn comparisons of the vB_EfaH_163 genome revealed the iF6, EfV12-phi1, and EFDG1 phage genomes to be the most similar (98.88%, 96.82%, and 95.75% similarity, respectively). These three phages belong to the *Herelleviriade*, subfamily *Brockvirinae*, genus *Schiekvirus*. The members of this family are characterised by their typical myovirus morphology, isometric head with rigid contractile tail, and the strong similarity of their genomes [54].

**Figure 4:**
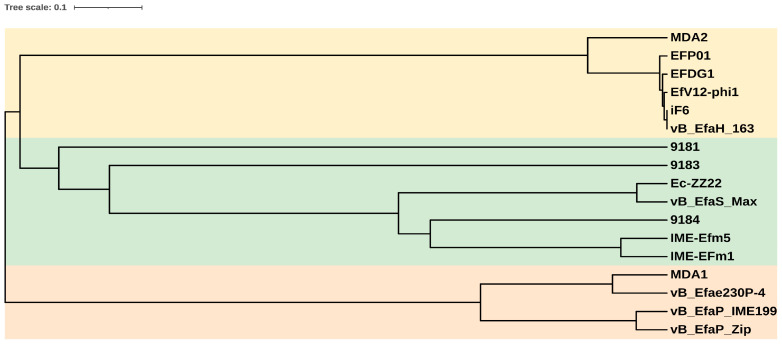
Phylogenetic tree of the *E. faecium* phages with complete genomes in databases, based on the amino acidic sequence of the major capsid proteins. Light yellow: *Herelleviridae* phages; light green: *Siphoviridae* phages; light orange: *Podoviridae* phages.

A phylogenetic tree based on the major capsid protein of the 17 *E. faecium*-infecting phages for which complete genomes are available in databases (Table 2) was made. Three clusters are visible (Figure 4). The vB_EfaH_163 phage grouped with five phages belonging to *Herelleviridae*, a second group was formed by *Siphoviridae* phages, and a third cluster by *Podoviridae* phages. Within the *Herelleviridae* phages, the phage MDA2 clustered in a different branch than the others, as it is classified as *Kochikohdavirus* [44], whereas the others are classified as belonging to *Schiekvirus* genus. The morphological characteristics, the similarity with the iF6, EfV12-phi1, and EFDG1 genomes, and the phylogenetic tree, all suggested phage vB_EfaH_163 to be a new member of *Herelleviriade*, subfamily *Brockvirinae*. Moreover, based on a higher than 95% similarity with the above mentioned phage genomes [55], the vB_EfaH_163 can be assumed to belong to the same genus, *Schiekvirus*.

### 3.4. Technological Characterisation

**Figure 5:**
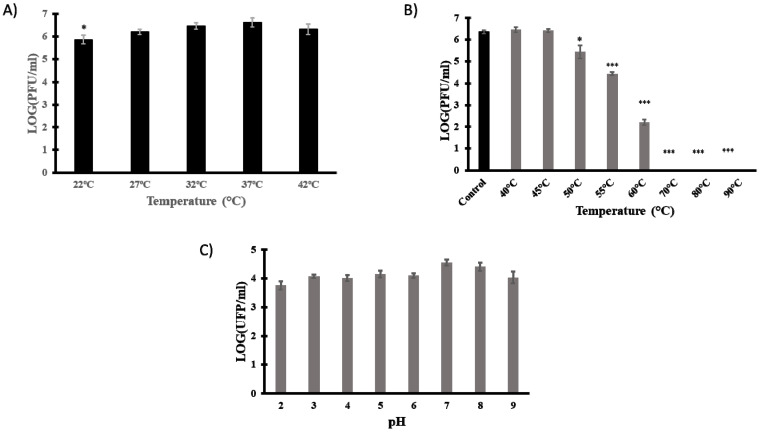
Technological characterisation of phage vB_EfmH_163. The effect of incubation temperature (22, 27, 32, 37, and 42 °C) (**A**), thermal stability (15 min-treatment at 40, 45, 50, 55, 60, 70, 80, or 90 °C) (**B**) and pH stability (15 min treatment in PBS pH 2–9) (**C**) were assessed by measuring the phage titre (Log PFU/mL). Means and standard deviations of three independent replicates are shown. *: *p* < 0.05; ***: *p* < 0.001.

Phages intended to be used as therapeutic agents must be able to withstand environmental temperatures and pHs. The effect of temperature on vB_EfaH_163 infection was therefore examined. Phage titre determinations were made in double-agar plates incubated at different temperatures (22, 27, 32, 37, and 42 °C). No significant differences were observed between plaque titres at 27, 32, 37, and 42 °C, but small differences were detected when the plates were incubated at 22 °C (Figure 5A).

Thermal and pH stability were then investigated. Phage titre determinations were made after 15 min incubation at different temperatures (room temperature, 40, 45, 50, 55, 60, 70, 80, and 90 °C) or different pHs (2, 3, 4, 5, 6, 7, 8, and 9). The phage titre did not significantly change at temperatures below 45 °C, but reductions of 1 log PFU/mL, 2 log PFU/mL, and 3 log PFU/mL were observed at 50, 55, and 60 °C, respectively. At temperatures above 70 °C, phage viability was lost (Figure 5B). Finally, given the slight and non-significant reduction in titre at all pHs tested (control pH = 7), phage vB_EfaH_163 can be deemed stable over the pH range studied (Figure 5C).

### 3.5. Biocontrol of E. faecium VR-13 by vB_EfaH_163

**Figure 6:**
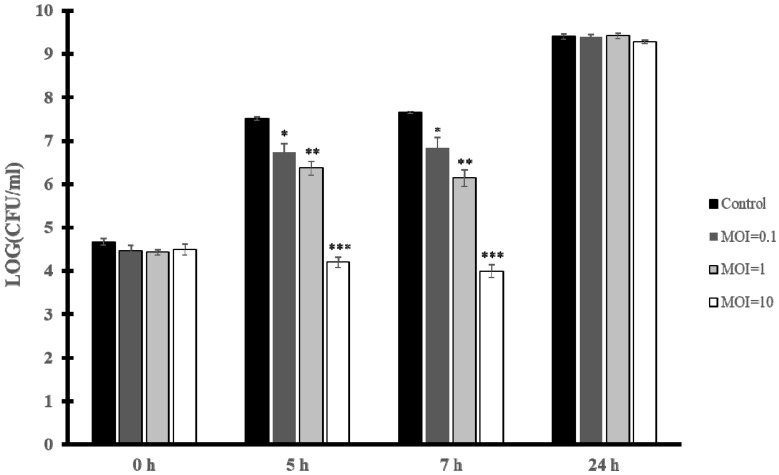
Biocontrol assay. *E. faecium* VR-13 10^4^ CFU/mL culture infected with phage vB_EfmH_163 at different MOIs (0.1, 1, and 10). Viable cells (CFU/mL) were counted at 5 h, 7 h, and 24 h post-infection. Means and standard deviations of three independent replicates are shown. *: *p* < 0.05; **: *p* < 0.01; ***: *p* < 0.001.

To test the capacity of vB_EfaH_163 to reduce the growth of *E. faecium*, biocontrol experiments were performed in culture broth with the VRE clinical strain *E. faecium* VR-13 as the target. An initial concentration of 10^4^ CFU/mL was incubated at 37 °C and after 1 h, phage vB_EfaH_163 was added at an MOI of 0.1, 1, or 10 and bacterial growth was measured as viable cell counts. A significant reduction in the number of the viable cells was observed after 5 h and 7 h for all the MOIs assayed (Figure 6).

The reduction in the number of cells was greater the more phage particles were present in the assay (Figure 6). At 7 h post-infection a reduction of 1 log(CFU/mL) in viable cells was seen at MOI = 0.1, of 2 log(CFU/mL) at MOI = 1, and 4 log(CFU/mL) at MOI = 10. This confirms vB_EfaH_163 to show biocontrol capacity for a period lasting at least 7 h post-infection, although at 24 h the *E. faecium* VR-13 strain was able to grow to a concentration similar to that reached by the control (Figure 6).

### 3.6. Reduction in the Mortality of Galleria mellonella Infected by E. faecium VR-13 due to Treatment with Phage vB_EfaH_163

**Figure 7:**
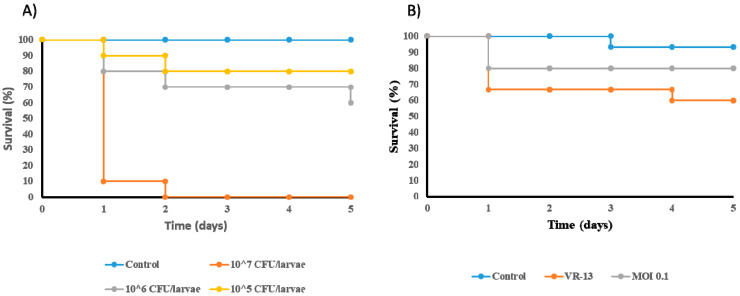
In vivo experiments in *Galleria mellonella*. A suitable concentration of *E. faecium* VR-13 per larvae was determined. Control larvae were inoculated with 10 μl of PBS. (**A**) and the effect of the treatment with phage vB_EfmH_163 at MOI = 0.1 (**B**) tested. Control larvae were inoculated with 10 μl of dH_2_O. N = 10 for each experiment and condition.

The potential of vB_EfaH_163 to combat VRE *E. faecium* was also tested in a *G. mellonella* model. First, the lethality of VRE *E. faecium* VR-13 was determined by injecting moth larvae with concentrations of 10^5^, 10^6^, or 10^7^ CFU of bacteria (PBS was used as a control). Larval survival was monitored for five days after infection (Figure 7A). After 48 h, all larvae injected with 10^7^ CFU/larvae had died. After five days, mortalities of 20% and 30% were recorded for inoculum concentrations of 10^5^ and 10^6^ CFU/larvae, respectively.

A concentration of 10^6^ CFU/larvae was selected for the challenge test with phage vB_EfaH_163 at MOI = 0.1 (Figure 7B). Infection with *E. faecium* VR-13 killed about 40% of the larvae after five days. Treatment with phage vB_EfaH_163 at MOI = 0.1 increased larval survival by 20%, although the observed differences were not statistically significant (Figure 7B).

## 4. Discussion

Vancomycin-resistance in *E. faecium* isolates is increasing [6,11,13]; new alternatives to combat infections caused by these bacteria are much needed [4]. Indeed, *E. faecium* is on the WHO’s priority list of ESKAPE microorganisms for which new therapies are required [11]. Phage therapy is a candidate. Phages are obligate parasites of bacteria found wherever their hosts are present [56]. Although enterococci have been documented in many different ecosystems, most of the phages that infect clinically important *E. faecium* strains have been isolated from sewage water. The present work explores human faecal samples as a source of new *E. faecium*-infecting phages with applications in phage therapy. Human faecal samples are complex matrices to work with, but the method designed by [31], with slight modifications, allowed samples to be easily cleaned and used in enrichment cultures. As a result, phage vB_EfaH_163 was isolated and further characterised. Host range analysis showed that this phage infects many *E. faecium* strains (51% of those tested; Table 1); this contrasts with the narrow host range described for phages of lactic acid bacteria, which is sometimes limited to just one strain [57], including those infecting Enterococci species [33]. The host range of vB_EfaH_163 includes 16 VRE *E. faecium* clinical isolates. Another phage able to infect VRE strains has been described, however its host range is narrower and limited to *E. faecalis* [58]. In this sense, it is noteworthy than in addition to *E. faecium* strains, the phage vB_EfaH_163 was able to infect two strains of *E. faecalis* (Table 1), one of them resistant to linezolid, a last resort antibiotic [14]. Although a rare event, other phages infecting *E. faecium* have been shown to infect some *E. faecalis* strains [4,20,44]. *E. faecalis* is the second most important species of *Enterococcus* that causes problematic nosocomial infections. Both species are similar in their microbiology and in the diseases they cause [11]. A phage that could be used to treat infections caused by both species would be a great boon.

The genome of vB_EfaH_163 is quite large at 150,836 bp. This is within the range of other *E. faecium*-infecting phages belonging to the *Herelleviridae* family, such as the 147,589 bp genome of EDFG1 [20] or the 156,592 bp genome of IF6 (Table 2). Most of the proteins encoded were annotated as hypothetical, with no putative function assigned. This is not uncommon when phage genomes are annotated; usually only a small percentage of *orf* products show homology with proteins available in databases, a consequence of wide bacteriophage diversity and the low levels of proteins characterised [59]. Those proteins with a putative function clustered into functional modules: packaging, lysis, structural (subclustered into head and tail proteins), and replication. This cluster organization might facilitate coordinated transcription, and could promote phage genome diversity via the exchange of similarly organised functional modules. Two putative lysins with similar catalytic domains (corresponding to muramidase activities) were encoded by the genome of vB_EfaH_163, (Supplementary Table S1; Figure 3). The structural module is composed of genes that code for several proteins similar to others of known function, such as portal protein, head mature protease, major capsid protein, tail sheath, and tail tape measure, and baseplate structure proteins (Supplementary Table S1; Figure 3). Interestingly, the encoding of a carbohydrate binding protein (Orf 28) next to a tail protein with a hydrolase domain (Orf 27) (Supplementary Table S1) indicates that both may be involved in the entry of viral DNA (via the recognition of a structure on the bacterial surface and the hydrolysis of the cell wall). Proteins with hydrolase domains at their tips, together with endolysins, have been proposed as antimicrobial enzybiotics against AMR bacteria [60]. In the opposite orientation to most of these functional modules, up to 21 *tRNA* genes were found lying close to other encoding proteins predicted to be involved in RNA metabolism (Supplementary Table S1; Figure 3). Although this seems to be a common feature in members of the *Brockvirinae* subfamily, their role in cell infection has not been established.

The predicted packaging system for vB_EfaH_163 (long direct terminal repeats) involves the recognition of specific sequences by the terminase, if phage DNA is to be encapsidated. This packaging system reduces the possibility of the undesirable host transduction of AMR, or virulence genes. In fact, no virulence factors, including toxin-encoding genes or antibiotic-resistance genes, were detected in the phage genome; it might, therefore, be safely used in phage therapy, although safety trials should be performed first.

A BLASTn comparison of the full phage genome revealed it to be very similar to those of the *E. faecium* phages EFDG1, IF6, and EfV12-phi1 (data not shown). These two phages and vB_EfaH_163 clustered with phages belonging to the family *Herelleviridae*, subfamily *Brockvirinae*, and genus *Schiekvirus* (Figure 4; Table 2). Members of the subfamily *Brockvirinae* are defined by a series of morphological and genomic characteristics: (1) all have a myovirus morphology with an isometric, icosahedral head joined to a uncontracted tail and a baseplate; (2) their genome is within the range of 125–170 kpb, it harbours a series of *tRNA* encoding genes, and most of the coding genes it contains are transcribed in the same direction; and (3) they have terminal repeat regions as a recognition sequence for DNA packaging [54]. All these characteristics are fulfilled by phage vB_EfaH_163: not only does it show typical myovirus morphology (Figure 2); it has a large genome of 150,836 bp (within the observed range of *Brockvirinae* subfamily members) with a series of 21 *tRNA* genes transcribed in the opposite direction to most of the genes encoded in the genome (Figure 3; Supplementary Table S1); and it has direct terminal repeats as predicted by the Phageterm tool. These characteristics, and its similarity to other *Herelleviridae* phages, classify phage vB_EfaH_163 within *Herelleviridae*, subfamily *Brockvirinae*, and genus *Schiekvirus*.

To our knowledge, neither the latent period nor burst size have been reported for the other *E. faecium* phages of the *Herelleviridae* family. Compared with *E. faecium* phages belonging to other families, the observed latent period for vB_EfaH_163 was high at 60 min compared, for example, with 10 min for the *E. faecium* Max phage [4]. However, vB_EfaH_163 has a much larger burst size (155 phages/infected bacterium) compared with the 38 phages/infected bacterium for Max [4], or the 60 phages/infected bacterium of phage IME-EF1 [61]. A larger burst size results in a larger number of phages that can infect a larger number of bacteria. However, the therapeutic success of a phage with a large burst size cannot be predicted [62].

To further assess the therapeutic potential of vB_EfaH_163, virion stability at different temperatures and pHs were tested. No significant difference was seen in terms of PFU/mL when incubations were performed within the range of human body temperature, not even at high fever temperatures. Therefore, it could also be useful in patients with fever. Moreover, this wide range of temperature could allow other future applications related to food safety, a field in which *E. faecium* is also relevant due to its ability to produce toxic biogenic amines [28,63]. In fact, the phage was active up to 60 °C, similar to the temperature stability described for the Max and Zip phages [4]. It was also stable over a wide range of pH values (from 2 to 9). Max and Zip phage stability is greatly reduced below pH 4, and undetectable at pH 3 for Zip, and at pH 2 for Max [4]. vB_EfaH_163 is also physicochemically stable over a wider range than other *E. faecium* phages. Together, these results confirm it to show promise for use in phage therapy.

The functionality of vB_EfaH_163 against clinical *E. faecium* isolates was tested against VRE *E. faecium* VR-13 isolated at the *Hospital del Bierzo* in northern Spain, a region with a high prevalence of VRE. Challenge experiments in broth revealed vB_EfaH_163 capable of reducing the growth of this bacterium in a concentration-dependent manner (Figure 6). However, the effect was temporary; strong growth inhibition was observed at 5–7 h, but the bacterium recovered and grew again at 24 h (Figure 6). The observed re-growth of the bacteria after 24 h could be considered a limitation for phage therapy. If the phage does not kill all the *E. faecium* cells during the treatment, a re-emergence of the infection could occur. This is a general drawback of phage therapy, and larger treatment periods, the use of phage cocktails, or a combination with antibiotic treatment have been suggested as possible solutions to improve effectiveness [7,45].

Although the *G. mellonella* animal model has been used to examine the virulence of VRE enterococci [64], the effectiveness of different antimicrobial treatments [65], and the potential of phage therapy against pathogens such as *Staphylococcus aureus* [66] and different Gram-negative species [50], it has not usually been employed for assessing the therapeutic potential of a phage infecting VRE *E. faecium* strains, although some reports can be found ([44]; Figure 7A). The virulence of this bacterium is similar to that reported for other VRE enterococcal strains of clinical origin [52,65]. The treatment of infected *G. mellonella* larvae with vB_EfaH_163 resulted in reduced mortality, reaching values similar to the uninfected control.

The *G. mellonella* model is a cost-effective model that can be used to evaluate the potential of phage therapy, including single phage, phage cocktail, and phage-antibiotic treatments, before scaling up to murine models. Previous comparative experiments have returned similar results with both types of models [51].

In summary, this work explores an alternative source, human stools, for screening for phages that can infect VRE enterococci. Phage vB_EfaH_163, a new member of *Herelleviridae*, subfamily *Brockvirinae*, and genus *Schiekvirus* shows features that render it a candidate for therapeutic use. It has a large host range, including clinical VRE *E. faecium* strains, and can even infect some *E. faecalis* strains of health concern, such as those resistant to last resort antibiotics. The phage’s packaging method reduces the chances of transduction, and no virulence or antibiotic resistance genes were found in its genome. It shows good virion stability and the capacity to control the growth of VRE *E. faecium* VR-13, both in broth and in a *G. mellonella* infection model. vB_EfaH_163 could increase phage therapy options for combating VRE enterococci, although safety and effectiveness studies should be performed first.

## Figures and Tables

**Table 1:** *E. faecium* and *E. faecalis* strains used in this work for bacteriophage vB_EfmH_163 isolation and host range determination.

Specie	Strain	Origin	vB_EfmH_163 Infection	Reference
*E. faecium*	LMA2	Camel milk	**+**	MicroMol
*E. faecium*	LMA3	Camel milk	**+**	MicroMol
*E. faecium*	LMA4	Camel milk	**+**	MicroMol
*E. faecium*	LMA5	Camel milk	**+**	MicroMol
*E. faecium*	LMA6	Camel milk	**+**	MicroMol
*E. faecium*	LMA8	Camel milk	**+**	MicroMol
*E. faecium*	LMA9	Camel milk	**−**	MicroMol
*E. faecium*	LMA10	Camel milk	**+**	MicroMol
*E. faecium*	LGMY-2	Camel milk	**+**	MicroMol
*E. faecium*	LGMY-5	Camel milk	**+**	MicroMol
*E. faecium*	LGMY-1	Cow milk	**+**	MicroMol
*E. faecium*	LMGY-10	Cow milk	**−**	MicroMol
*E. faecium*	LGMY-6	Sheep milk	**+**	MicroMol
*E. faecium*	LGMY-11	Goat milk	**+**	MicroMol
*E. faecium*	C39	Cheese	**−**	[28]
*E. faecium*	AM	Cheese	**−**	[28]
*E. faecium*	103	Cheese	**−**	[28]
*E. faecium*	LGMY-12	Date	**+**	MicroMol
*E. faecium*	LMGY-13	Date	**+**	LGM
*E. faecium*	LGM11397	Meat	**−**	LGM
*E. faecium*	LGM14205	Meat	**−**	LGM
*E. faecium*	LGM20641	Meat	**−**	LGM
*E. faecium*	HF11	Human	**−**	[29]
*E. faecium*	HF14	Human	**+**	[29]
*E. faecium*	HF24	Human	**+**	[29]
*E. faecium*	HF52	Human	**−**	[29]
*E. faecium*	HF56	Human	**+**	[29]
*E. faecium*	VR-1	Clinical	**−**	Bierzo Hospital
*E. faecium*	VR-2	Clinical	**−**	Bierzo Hospital
*E. faecium*	VR-3	Clinical	**−**	Bierzo Hospital
*E. faecium*	VR-4	Clinical	**−**	Bierzo Hospital
*E. faecium*	VR-5	Clinical	**−**	Bierzo Hospital
*E. faecium*	VR-6	Clinical	**−**	Bierzo Hospital
*E. faecium*	VR-7	Clinical	**+**	Bierzo Hospital
*E. faecium*	VR-8	Clinical	**+**	Bierzo Hospital
*E. faecium*	VR-9	Clinical	**−**	Bierzo Hospital
*E. faecium*	VR-10	Clinical	**+**	Bierzo Hospital
*E. faecium*	VR-11	Clinical	**−**	Bierzo Hospital
*E. faecium*	VR-12	Clinical	**−**	Bierzo Hospital
*E. faecium*	VR-13	Clinical	**+**	Bierzo Hospital
*E. faecium*	VR-14	Clinical	**+**	Bierzo Hospital
*E. faecium*	VR-15	Clinical	**−**	Bierzo Hospital
*E. faecium*	VR-16	Clinical	**+**	Bierzo Hospital
*E. faecium*	VR-17	Clinical	**−**	Bierzo Hospital
*E. faecium*	VR-18	Clinical	**+**	Bierzo Hospital
*E. faecium*	VR-19	Clinical	**−**	Bierzo Hospital
*E. faecium*	VR-20	Clinical	**−**	Bierzo Hospital
*E. faecium*	VR-20b	Clinical	**+**	Bierzo Hospital
*E. faecium*	VR-22	Clinical	**+**	Bierzo Hospital
*E. faecium*	VR-23	Clinical	**+**	Bierzo Hospital
*E. faecium*	VR-24	Clinical	**−**	Bierzo Hospital
*E. faecium*	VR-25	Clinical	**+**	Bierzo Hospital
*E. faecium*	VR-26	Clinical	**+**	Bierzo Hospital
*E. faecium*	VR-27	Clinical	**+**	Bierzo Hospital
*E. faecium*	VR-28	Clinical	**−**	Bierzo Hospital
*E. faecium*	VR-29	Clinical	**−**	Bierzo Hospital
*E. faecium*	VR-30	Clinical	**+**	Bierzo Hospital
*E. faecium*	VR-31	Clinical	**−**	Bierzo Hospital
*E. faecium*	VR-32	Clinical	**+**	Bierzo Hospital
*E. faecium*	VR-33	Clinical	**−**	Bierzo Hospital
*E. faecium*	VR-34	Clinical	**+**	Bierzo Hospital
*E. faecium*	VR-35	Clinical	**−**	Bierzo Hospital
*E. faecium*	VR-36	Clinical	**−**	Bierzo Hospital
*E. faecium*	VR-37	Clinical	**−**	Bierzo Hospital
*E. faecium*	VR-38	Clinical	**−**	Bierzo Hospital
*E. faecium*	VR-39	Clinical	**−**	Bierzo Hospital
*E. faecium*	VR-40	Clinical	**−**	Bierzo Hospital
*E. faecium*	VR-41	Clinical	**−**	Bierzo Hospital
*E. faecium*	VR-42	Clinical	**−**	Bierzo Hospital
*E. faecium*	VR-43	Clinical	**−**	Bierzo Hospital
*E. faecium*	VR-44	Clinical	**−**	Bierzo Hospital
*E. faecium*	VR-45	Clinical	**−**	Bierzo Hospital
*E. faecium*	VR-46	Clinical	**−**	Bierzo Hospital
*E. faecium*	VR-47	Clinical	**−**	Bierzo Hospital
*E. faecium*	VR-48	Clinical	**−**	Bierzo Hospital
*E. faecium*	VR-49	Clinical	**−**	Bierzo Hospital
*E. faecium*	VR-50	Clinical	**−**	Bierzo Hospital
*E. faecalis*	CECT481^T^	Type strain	**−**	CECT
*E. faecalis*	18a	Cheese	**−**	[28]
*E. faecalis*	23a	Cheese	**−**	[28]
*E. faecalis*	V63	Cheese	**−**	[28]
*E. faecalis*	63c	Cheese	**−**	[28]
*E. faecalis*	52c	Cheese	**−**	[28]
*E. faecalis*	HFS56	Human	**+**	[29]
*E. faecalis*	HFS57	Human	**−**	[29]
*E. faecalis*	VR-5	Clinical	**−**	Bierzo Hospital
*E. faecalis*	VR-11	Clinical	**−**	Bierzo Hospital
*E. faecalis*	optra5	Clinical	**+**	Bierzo Hospital
*E. faecalis*	V583	Clinical	**−**	[30]

Bierzo Hospital: Strain isolated at the Microbiology unit at the El Bierzo Hospital, Spain; CECT: *Colección Española de Cultivos Tipo*; LMG: *Laboratorium voor Microbiologie*; MicroMol: Culture collection of the Molecular Microbiology Group at IPLA. Clinical origin refers to strains isolated from humans in a clinical ambient. Human origin refers to strains isolated from humans outside a clinical environment.

**Table 2:** Characteristics of the *E. faecium* phages used in the phylogenetic analysis. Genome size and accession number are shown.

Phage	Accession Number	Host	Family	Genome Size	Origin	Reference
vB_EfmH_163	CAJDKA010000002.1	*E. faecium* *E. faecalis*	*Herelleviridae*	150,836	Human faecal samples	This work
EFDG1	NC_029009	*E. faecium* *E faecalis*	*Herelleviridae*	147,589	Sewage effluents	[21]
EfV12-phi1	MH880817	*E. faecium*	*Herelleviridae*	152,770	Sewage	[43]
EFP01	NC_047796.1	*E. faecium*	*Herelleviridae*	155,053	Sewage	-
iF6	MT909815.1	*E. faecium*	*Herelleviridae*	156,592	-	-
MDA2	MW633168.1	*E. faecium*	*Herelleviridae*	140,226	-	[44]
9183	MT939241.1	*E. faecium*	*Siphoviridae*	806,301	Wastewater	[45]
9181	MT939240.1	*E. faecium*	*Siphoviridae*	71,854	Wastewater	[45]
vB_EfaS_Max	MK360024	*E. faecium* *E. faecalis*	*Siphoviridae*	40,975	Raw sewage from wastewater	[4]
9184	MT939242.1	*E. faecium*	*Siphoviridae*	44,108	Wastewater	[45]
Ec-ZZ2	NC_031260	*E. faecium*	*Siphoviridae*	41,170	Sewage	[46]
IME-EFm1	NC_024356	*E. faecium*	*Siphoviridae*	42,597	Hospital sewage	[47]
IME-EFm5	NC_028826	*E* *. faecium*	*Siphoviridae*	42,265	Hospital sewage	[48]
vB_EfaP_Zip	MK360025	*E. faecium* *E. faecalis*	*Podoviridae*	18,742	Raw sewage from wastewater	[4]
vB_Efae230p-4	NC_025467	*E. faecium*	*Podoviridae*	17,972	-	-
vB_EfaP_IME199	KT945995	*E. faecium*	*Podoviridae*	18,838	Sewage	[49]
MDA1	MW623430.1	*E. faecium*	*Podoviridae*	18,058	-	[44]

-: information not available.

## Data Availability

Publicly available sequence dataset generated in this study can be in the European Nucleotide Archive (ENA) under accession number CAJDKA010000002.1.

## Supplementary Materials

The following are available online at https://www.mdpi.com/article/10.3390/v15010179/s1, Table S1: Genome features of the *Enterococcus faecium* bacteriophage vB_EfaH_163.
