# Supplementary material for: Enterococcus faecium Bacteriophage vB_EfaH_163, a New Member of the Herelleviridae Family, Reduces the Mortality Associated with an E. faecium vanR Clinical Isolate in a Galleria mellonella Animal Model
Source: Viruses. 2023 Jan 7;15(1):179. doi: 10.3390/v15010179 (PMC9860891; doi:10.3390/v15010179)
Supplement: Supplementary file 1 [file viruses-15-00179-s001.zip › Supplementary Material Table S1.pdf]

Supplementary Material

Enterococcus faecium Bacteriophage vB_EfaH_163, a New Member of the *Herelleviridae* Family, Reduces the Mortality Associated with an *E. faecium* vanR Clinical Isolate in a *Galleria mellonella* Animal Model

Inés Pradal [1], Angel Casado [1], Beatriz del Rio [1,2,], Carlos Rodriguez-Lucas [2,3,4], Maria Fernandez [1,2], Miguel A. Alvarez [1,2] and Victor Ladero [1,2,*]

[1] Department of Technology and Biotechnology of Dairy Products, Dairy Research Institute, IPLA-CSIC, Villaviciosa 33300, Spain
[2] Instituto de Investigación Sanitaria del Principado de Asturias (ISPA), Oviedo 33011, Spain
[3] Microbiology Laboratory, Hospital el Bierzo, Ponferrada 24404, Spain
[4] Microbiology Laboratory, Hospital Universitario de Cabueñes, Gijón 33394, Spain
* Correspondence: ladero@ipla.csic.es; Tel.: +00-34-985-89-33-59

Supplementary Table S1: Features of *Enterococcus faecium* bacteriophage vB_EfaH_163. The *orfs*, gene number and gene position in the vB_EfaH_163 genome are shown, as are the predicted functions, molecular weights and isoelectric points of the encoded products. The predicted functions were assessed using RAST and PATRIC software. The top BLAST hit and E-values are also indicated.

ORF	Gene	Start (bp)	Stop (bp)	Length (aa)	MW (kDa)	IP	Predicted function	BLAST_HIT	E-VALUE
1	vB_EfaH_163-001	55	324	89	10.27	9.84	hypothetical protein	putative membrane protein [Enterococcus phage iF6]	1.00E-55
2	vB_EfaH_163-002	344	622	92	10.38	9.8	hypothetical phage protein	hypothetical protein HOR47_gp104 [Enterococcus phage EFP01]	9.00E-61
3	vB_EfaH_163-003	627	1055	142	16.01	4.39	hypothetical phage protein	hypothetical protein AVV19_p32 [Enterococcus phage EFDG1]	3.00E-96
4	vB_EfaH_163-004	1055	2890	611	69.45	6.97	Phage terminase, large subunit	terminase large subunit [Enterococcus phage vB_OCPT_Ben]	0.00E+00
5	vB_EfaH_163-005	3134	3979	281	31.12	5.11	hypothetical phage protein	hypothetical protein PEf771_55 [Enterococcus phage PEf771]	0.00E+00

6	vB_EfaH_163-006	3976	4803	275	31.89	4.74	hypothetical phage protein	hypothetical protein AVV19_gp124 [Enterococcus phage EFDG1]	0.00E+00
7	vB_EfaH_163-007	4804	5160	118	13.09	4.99	hypothetical protein	hypothetical protein iF6_81 [Enterococcus phage iF6]	5.00E-80
8	vB_EfaH_163-008	5262	6530	422	46.26	9.43	N-acetylmuramoyl-L-alanine amidase	N-acetylmuramoyl-L-alanine amidase [Enterococcus phage iF6]	0.00E+00
9	vB_EfaH_163-009	6688	7326	212	23.00	4.36	Aggregation promoting factor	aggregation promoting factor [Enterococcus phage EFDG1]	7.00E-138
10	vB_EfaH_163-010	7430	8374	314	34.11	9.69	N-acetylmuramoyl-L-alanine amidase	N-acetylmuramoyl-L-alanine amidase [Enterococcus phage EFDG1]	0.00E+00
11	vB_EfaH_163-011	8504	8848	114	13.24	5.51	hypothetical phage protein	hypothetical protein AVV19_gp119 [Enterococcus phage EFDG1]	4.00E-78
12	vB_EfaH_163-012	8865	10553	562	62.99	5.97	Portal protein	portal protein [Enterococcus phage iF6]	0.00E+00
13	vB_EfaH_163-013	10653	11441	262	29.55	5.02	Prohead protease	prohead protease [Enterococcus phage EFDG1]	0.00E+00
14	vB_EfaH_163-014	11441	12445	334	37.47	4.21	hypothetical protein	hypothetical protein iF6_88 [Enterococcus phage iF6]	0.00E+00
15	vB_EfaH_163-015	12568	13989	473	52.20	5.28	Major capsid protein	major capsid protein [Enterococcus phage iF6]	0.00E+00
16	vB_EfaH_163-016	14087	14338	83	9.42	9.37	hypothetical protein	hypothetical protein AVV19_p30 [Enterococcus phage EFDG1]	1.00E-48
17	vB_EfaH_163-017	14350	15246	298	33.45	4.97	Head structural protein	head completion protein [Enterococcus phage iF6]	0.00E+00
18	vB_EfaH_163-018	15260	16132	290	32.94	6.66	hypothetical phage protein	hypothetical protein iF6_92 [Enterococcus phage iF6]	0.00E+00
19	vB_EfaH_163-019	16125	16748	207	23.85	10.28	hypothetical phage protein	hypothetical protein AVV19_gp109 [Enterococcus phage EFDG1]	3.00E-150
20	vB_EfaH_163-020	16752	17597	281	31.93	4.83	hypothetical phage protein	hypothetical protein HOR47_gp081 [Enterococcus phage EFP01]	0.00E+00
21	vB_EfaH_163-021	17597	17836	79	9.09	9.27	hypothetical phage protein	hypothetical protein HOR47_gp080 [Enterococcus phage EFP01]	5.00E-49
22	vB_EfaH_163-022	17840	19549	569	62.06	5.04	Phage tail sheath	major tail sheath protein [Enterococcus phage EFP01]	0.00E+00
23	vB_EfaH_163-023	19615	20037	140	15.49	5.37	Phage tail protein	tail tube protein [Enterococcus phage EFDG1]	2.00E-97
24	vB_EfaH_163-024	20295	20768	157	18.44	5.17	hypothetical phage protein	hypothetical protein HOR47_gp076 [Enterococcus phage EFP01]	5.00E-112
25	vB_EfaH_163-025	20851	21417	188	21.82	4.37	hypothetical phage protein	hypothetical protein iF6_100 [Enterococcus phage iF6]	1.00E-131
26	vB_EfaH_163-026	21467	25099	1210	129.88	8.54	Phage tail tape measure	putative tail lysin [Enterococcus phage EFP01]	0.00E+00
27	vB_EfaH_163-027	25149	27668	839	94.11	4.82	Tail protein with hydrolase domain	repressor protein C2 [Enterococcus phage EFP01]	0.00E+00
28	vB_EfaH_163-028	27768	34805	2345	260.05	4.83	Carbohydrate binding domain protein	putative central tail fibre [Enterococcus phage iF6]	0.00E+00
29	vB_EfaH_163-029	34895	37306	803	88.11	8.83	hypothetical phage protein	DUF859 domain-containing protein [Enterococcus phage iF6]	0.00E+00
30	vB_EfaH_163-030	37303	38046	247	28.54	4.58	hypothetical protein	hypothetical protein iF6_105 [Enterococcus phage iF6]	2.00E-177
31	vB_EfaH_163-031	38067	38213	48	5.70	5.04	hypothetical phage protein	hypothetical protein iF6_106 [Enterococcus phage iF6]	5.00E-25
32	vB_EfaH_163-032	38368	39069	233	25.95	8.6	Phage tail protein	tail tube terminator protein [Enterococcus phage iF6]	8.00E-171
33	vB_EfaH_163-033	39069	39605	178	20.38	4.93	hypothetical phage protein	hypothetical protein AVV19_gp094 [Enterococcus phage EFDG1]	4.00E-126

34	vB_EfaH_163-034	39592	40296	234	26.41	4.78	Phage baseplate assembly protein	baseplate assembly protein [Enterococcus phage iF6]	7.00E-172
35	vB_EfaH_163-035	40311	41363	350	39.46	5.28	Phage baseplate protein	putative baseplate J protein [Enterococcus phage EFP01]	0.00E+00
36	vB_EfaH_163-036	41375	45016	1213	136.47	5.04	Phage baseplate assembly protein	baseplate assembly protein [Enterococcus phage iF6]	0.00E+00
37	vB_EfaH_163-037	45031	47565	844	93.57	5.52	hypothetical protein	hypothetical protein EfsSzw1_124 [Enterococcus phage EfsSzw-1]	0.00E+00
38	vB_EfaH_163-038	47581	48090	169	18.71	4.26	hypothetical protein	hypothetical protein EfsSzw1_125 [Enterococcus phage EfsSzw-1]	2.00E-93
39	vB_EfaH_163-039	48186	48731	181	20.33	5.56	Phage baseplate assembly protein	baseplate assembly protein [Enterococcus phage iF6]	8.00E-132
40	vB_EfaH_163-040	48748	52206	1152	129.18	4.84	Putative adsorption tail protein	putative adsorption associated tail protein [Enterococcus phage EFP01]	0.00E+00
41	vB_EfaH_163-041	52287	53249	320	35.99	9.49	hypothetical phage protein	hypothetical protein HOU42_gp144 [Enterococcus phage EfV12-phi1]	0.00E+00
42	vB_EfaH_163-042	53327	53533	68	7.97	9.63	hypothetical protein	hypothetical protein AVV19_gp086 [Enterococcus phage EFDG1]	1.00E-39
43	vB_EfaH_163-043	53727	55502	591	66.93	6.57	Phage DNA helicase	DNA helicase [Enterococcus phage EFGrKN]	0.00E+00
44	vB_EfaH_163-044	55518	57161	547	62.77	5.53	helix turn helix domain protein	putative transcriptional regulator [Enterococcus phage EFP01]	0.00E+00
45	vB_EfaH_163-045	57175	58647	490	55.31	5.02	Phage DNA helicase	DNA helicase [Enterococcus phage EFGrNG]	0.00E+00
46	vB_EfaH_163-046	58647	59687	346	39.08	5.02	Phage recombination exonuclease	DNA repair exonuclease SbcCD-like nuclease subunit [Enterococcus phage iF6]	0.00E+00
47	vB_EfaH_163-047	59806	61698	630	71.05	5.13	Phage recombination related exonuclease	recombination related exonuclease [Enterococcus phage EfV12-phi1]	0.00E+00
48	vB_EfaH_163-048	61712	62371	219	25.70	5.17	hypothetical phage protein	hypothetical protein iF6_122 [Enterococcus phage iF6]	1.00E-152
49	vB_EfaH_163-049	62375	63430	351	40.09	5.79	Phage DNA primase/helicase	DNA primase [Enterococcus phage iF6]	0.00E+00
50	vB_EfaH_163-050	63440	64321	293	33.22	5	dUTPase	dUTPase [Enterococcus phage iF6]	0.00E+00
51	vB_EfaH_163-051	64321	64557	78	8.91	4.98	hypothetical protein	hypothetical protein HOU42_gp134 [Enterococcus phage EfV12-phi1]	1.00E-48
52	vB_EfaH_163-052	64561	64872	103	11.57	4.81	hypothetical protein	hypothetical protein HOR47_gp050 [Enterococcus phage EFP01]	2.00E-67
53	vB_EfaH_163-053	64859	65170	103	11.75	4.34	hypothetical protein	putative phosphotransferase/anion transport protein [Enterococcus phage EFP01]	2.00E-65
54	vB_EfaH_163-054	65163	65531	122	14.19	5.8	hypothetical phage protein	hypothetical protein HOU42_gp131 [Enterococcus phage EfV12-	6.00E-82

								phi1]	
55	vB_EfaH_163-055	65533	66180	215	25.40	4.86	hypothetical protein	hypothetical protein PEf771_7 [Enterococcus phage PEf771]	2.00E-151
56	vB_EfaH_163-056	66173	66850	225	26.08	6.03	hypothetical phage protein	hypothetical protein [Enterococcus phage EFGrNG]	5.00E-166
57	vB_EfaH_163-057	66850	67215	121	13.88	4.06	hypothetical protein	hypothetical protein HOR47_gp043 [Enterococcus phage EFP01]	5.00E-77
58	vB_EfaH_163-058	67238	67375	45	4.94	4.12	hypothetical protein	hypothetical protein HOU42_gp127 [Enterococcus phage EfV12-phi1]	3.00E-23
59	vB_EfaH_163-059	67372	67794	140	16.11	6.3	hypothetical phage protein	DUF3310 domain-containing protein [Enterococcus phage iF6]	7.00E-101
60	vB_EfaH_163-060	67900	68694	264	31.40	7.65	hypothetical phage protein	hypothetical protein HOU42_gp125 [Enterococcus phage EfV12-phi1]	0.00E+00
61	vB_EfaH_163-061	68687	68998	103	11.99	9.43	Phage integration host factor	integration host factor [Enterococcus phage EFDG1]	4.00E-69
62	vB_EfaH_163-062	69080	69922	280	32.22	6.13	DNA polymerase I	putative DNA polymerase [Enterococcus phage vB_EfaM_A2]	0.00E+00
63	vB_EfaH_163-063	70093	70569	158	18.75	9.51	hypothetical phage protein	hypothetical protein AVV19_gp064 [Enterococcus phage EFDG1]	1.00E-111
64	vB_EfaH_163-064	70648	72837	729	83.84	5.88	DNA polymerase I	DNA polymerase I [Enterococcus phage EfV12-phi1]	0.00E+00
65	vB_EfaH_163-065	72960	73490	176	21.12	5.26	hypothetical phage protein	hypothetical protein AVV19_gp062 [Enterococcus phage EFDG1]	6.00E-125
66	vB_EfaH_163-066	73562	74878	438	48.25	4.82	hypothetical phage protein	hypothetical protein HOR47_gp031 [Enterococcus phage EFP01]	0.00E+00
67	vB_EfaH_163-067	74959	76206	415	46.08	5.51	Phage recombinase	recombinase [Enterococcus phage EfV12-phi1]	0.00E+00
68	vB_EfaH_163-068	76249	76623	124	14.18	6.73	hypothetical phage protein	hypothetical protein iF6_143 [Enterococcus phage iF6]	8.00E-88
69	vB_EfaH_163-069	76616	77233	205	23.96	9.3	hypothetical phage protein	hypothetical protein AVV19_gp057 [Enterococcus phage EFDG1]	1.00E-144
70	vB_EfaH_163-070	77301	77579	92	10.31	7.96	holin-like protein	holin-like protein [Enterococcus phage PEf771]	9.00E-61
71	vB_EfaH_163-071	77629	79074	481	50.58	4.63	hypothetical protein	putative Ig-like protein [Enterococcus phage EFP01]	0.00E+00
72	vB_EfaH_163-072	79101	79544	147	16.41	4.52	hypothetical phage protein	putative DNA polymerase [Enterococcus phage EFP01]	6.00E-100
73	vB_EfaH_163-073	79652	80056	134	15.61	9.1	hypothetical protein	putative transmembrane protein [Enterococcus phage vB_EfaM_A2]	1.00E-81
74	vB_EfaH_163-074	80152	80388	78	9.03	4.99	hypothetical protein	hypothetical protein HOR47_gp022 [Enterococcus phage EFP01]	1.00E-45
75	vB_EfaH_163-075	80381	80650	89	10.88	7.68	hypothetical protein	hypothetical protein A2_168 [Enterococcus phage vB_EfaM_A2]	8.00E-55
76	vB_EfaH_163-076	80625	81563	312	35.28	5.36	hypothetical protein	DnaJ-type Zn-binding domain containing RecJ-like exonuclease [Enterococcus phage iF6]	0.00E+00
77	vB_EfaH_163-077	81621	82904	427	49.00	6.23	hypothetical protein	Mre11-like nuclease [Enterococcus phage iF6]	0.00E+00
78	vB_EfaH_163-078	82917	83285	122	14.32	9.26	hypothetical protein	hypothetical protein HOR47_gp018 [Enterococcus phage EFP01]	3.00E-79
79	vB_EfaH_163-079	83330	83974	214	23.92	9.2	hypothetical phage protein	hypothetical protein HOR47_gp017 [Enterococcus phage EFP01]	2.00E-155
80	vB_EfaH_163-080	83971	84711	246	28.40	9.2	hypothetical phage protein	hypothetical protein AVV19_gp047 [Enterococcus phage EFDG1]	0.00E+00
81	vB_EfaH_163-081	84704	85207	167	19.20	10.21	hypothetical phage protein	hypothetical protein HOR47_gp015 [Enterococcus phage EFP01]	2.00E-118

82	vB_EfaH_163-082	85218	86084	288	31.72	4.89	hypothetical phage protein	hypothetical protein iF6_156 [Enterococcus phage iF6]	0.00E+00
83	vB_EfaH_163-083	86196	86744	182	20.54	4.32	hypothetical protein	hypothetical protein HOR47_gp013 [Enterococcus phage EFP01]	2.00E-128
84	vB_EfaH_163-084	86757	87605	282	33.01	5.34	Thioredoxin, phage-associated	hypothetical protein HOR47_gp012 [Enterococcus phage EFP01]	0.00E+00
85	vB_EfaH_163-085	87605	89287	560	65.39	8.56	hypothetical phage protein	hypothetical protein HOR47_gp011 [Enterococcus phage EFP01]	0.00E+00
86	vB_EfaH_163-086	89562	90257	231	26.28	4.85	hypothetical phage protein	hypothetical protein AVV19_gp039 [Enterococcus phage EFDG1]	1.00E-165
87	vB_EfaH_163-087	90270	90734	154	17.69	4.6	hypothetical phage protein	hypothetical protein iF6_161 [Enterococcus phage iF6]	1.00E-108
88	vB_EfaH_163-088	90822	91526	234	26.43	7.57	hypothetical protein	hypothetical protein HOR47_gp008 [Enterococcus phage EFP01]	3.00E-159
89	vB_EfaH_163-089	91540	92220	226	26.65	5.33	hypothetical protein	hypothetical protein iF6_162 [Enterococcus phage iF6]	8.00E-151
90	vB_EfaH_163-090	92234	92977	247	28.46	6.14	hypothetical protein	hypothetical protein iF6_163 [Enterococcus phage iF6]	2.00E-61
91	vB_EfaH_163-091	93046	93345	99	12.05	4.64	hypothetical protein	hypothetical protein iF6_164 [Enterococcus phage iF6]	1.00E-44
92	vB_EfaH_163-092	93414	95843	809	92.65	4.35	hypothetical protein	hypothetical protein iF6_165 [Enterococcus phage iF6]	0.00E+00
93	vB_EfaH_163-093	95905	96354	149	17.17	7.91	hypothetical protein	hypothetical protein [Enterococcus phage EFGrNG]	9.00E-98
94	vB_EfaH_163-094	96382	96942	186	20.58	6.12	hypothetical protein	hypothetical protein iF6_167 [Enterococcus phage iF6]	3.00E-107
95	vB_EfaH_163-095	97163	97399	78	91.07	7.98	hypothetical protein	hypothetical protein EfsSzw1_180 [Enterococcus phage EfsSzw-1]	3.00E-46
96	vB_EfaH_163-096	97461	97709	82	90.17	9.63	hypothetical protein	repressor [Enterococcus phage EFDG1]	2.00E-49
97	vB_EfaH_163-097	97742	98182	146	16.76	8.67	hypothetical protein	transcriptional regulator [Enterococcus phage iF6]	2.00E-101
98	vB_EfaH_163-098	98335	98466	43	5.22	4.87	hypothetical protein	hypothetical protein AVV19_gp029 [Enterococcus phage EFDG1]	3.00E-19
99	vB_EfaH_163-099	98577	98903	108	12.47	4.12	hypothetical protein	hypothetical protein iF6_171 [Enterococcus phage iF6]	6.00E-67
100	vB_EfaH_163-100	98917	99405	162	18.90	9.72	hypothetical protein	hypothetical protein [Enterococcus phage EFGrKN]	3.00E-110
101	vB_EfaH_163-101	99545	99994	149	17.25	4.24	hypothetical protein	hypothetical protein [Enterococcus phage 156]	6.00E-99
102	vB_EfaH_163-102	100422	100697	91	10.42	6.1	hypothetical protein	hypothetical protein HOR47_gp190 [Enterococcus phage EFP01]	4.00E-56
103	vB_EfaH_163-103	100825	100953	42	5.11	10.56	hypothetical protein	hypothetical protein EfsSzw1_1 [Enterococcus phage EfsSzw-1]	5.00E-16
104	vB_EfaH_163-104	101014	101202	62	7.28	9.76	hypothetical protein	hypothetical protein PEf771_160 [Enterococcus phage PEf771]	1.00E-31
105	vB_EfaH_163-105	101289	101579	96	11.20	8.39	hypothetical protein	hypothetical protein HOR47_gp188 [Enterococcus phage EFP01]	2.00E-52
106	vB_EfaH_163-106	101712	101951	79	9.48	4.16	hypothetical protein	hypothetical protein HOR47_gp187 [Enterococcus phage EFP01]	1.00E-44
107	vB_EfaH_163-107	102039	102293	84	9.78	9.76	hypothetical protein	hypothetical protein HOR47_gp186 [Enterococcus phage EFP01]	3.00E-51
108	vB_EfaH_163-108	102308	102685	125	14.59	4.08	hypothetical protein	hypothetical protein HOR47_gp185 [Enterococcus phage EFP01]	8.00E-75
109	vB_EfaH_163-109	102705	102878	57	7.08	9.94	hypothetical protein	hypothetical protein PEf771_155 [Enterococcus phage PEf771]	4.00E-30
110	vB_EfaH_163-110	103051	103290	79	9.53	4.44	hypothetical protein	hypothetical protein iF6_183 [Enterococcus phage iF6]	6.00E-48
111	vB_EfaH_163-111	103376	103759	127	14.89	4.08	hypothetical protein	hypothetical protein iF6_184 [Enterococcus phage iF6]	4.00E-80
112	vB_EfaH_163-112	103876	104154	92	10.75	7.86	hypothetical protein	hypothetical protein HOR47_gp182 [Enterococcus phage EFP01]	8.00E-59
113	vB_EfaH_163-113	104242	104655	137	15.78	6.15	hypothetical protein	hypothetical protein HOR47_gp181 [Enterococcus phage EFP01]	8.00E-90

114	vB_EfaH_163-114	104679	104870	63	7.50	5	hypothetical protein	-	-
115	vB_EfaH_163-115	105486	105641	51	5.84	4.13	hypothetical protein	hypothetical protein iF6_1 [Enterococcus phage iF6]	5.00E-27
116	vB_EfaH_163-116	105694	105960	88	9.92	4.28	hypothetical protein	hypothetical protein PHIEF17H_1350 [Enterococcus phage phiEF17H]	1.00E-47
117	vB_EfaH_163-117	105975	106217	80	9.39	7.95	hypothetical protein	hypothetical protein iF6_3 [Enterococcus phage iF6]	2.00E-46
118	vB_EfaH_163-118	106233	106553	106	12.25	8.75	hypothetical protein	hypothetical protein iF6_4 [Enterococcus phage iF6]	2.00E-66
119	vB_EfaH_163-119	106627	106767	46	5.15	8.93	hypothetical protein	hypothetical protein [Enterococcus phage EFGrKN]	4.00E-24
120	vB_EfaH_163-120	106857	107000	47	5.66	3.84	hypothetical protein	hypothetical protein iF6_6 [Enterococcus phage iF6]	2.00E-22
121	vB_EfaH_163-121	107405	107193	70	7.56	4.12	hypothetical protein	hypothetical protein iF6_7 [Enterococcus phage iF6]	6.00E-39
122	vB_EfaH_163-122	107733	107461	90	10.79	5.34	hypothetical protein	hypothetical protein HOR47_gp172 [Enterococcus phage EFP01]	2.00E-58
123	vB_EfaH_163-123	108161	107751	136	15.43	4.87	hypothetical protein	hypothetical protein HOR47_gp171 [Enterococcus phage EFP01]	2.00E-93
124	vB_EfaH_163-124	108399	108175	74	8.54	7.87	hypothetical protein	putative transmembrane protein [Enterococcus phage vB_EfaM_A2]	1.00E-42
125	vB_EfaH_163-125	108624	108400	74	8.36	7.73	hypothetical protein	hypothetical protein HOR47_gp169 [Enterococcus phage EFP01]	4.00E-44
126	vB_EfaH_163-126	108852	108625	75	8.73	4.46	hypothetical protein	hypothetical protein [Enterococcus phage vB_OCPT_Ben]	4.00E-45
127	vB_EfaH_163-127	109087	108854	77	9.17	4.14	hypothetical protein	hypothetical protein iF6_12 [Enterococcus phage iF6]	9.00E-45
128	vB_EfaH_163-128	109479	109174	101	11.83	5.42	hypothetical protein	hypothetical protein iF6_13 [Enterococcus phage iF6]	1.00E-58
129	vB_EfaH_163-129	109689	109492	65	7.71	9.74	hypothetical protein	hypothetical protein iF6_14 [Enterococcus phage iF6]	1.00E-38
130	vB_EfaH_163-130	109853	109689	54	6.35	3.94	hypothetical protein	hypothetical protein iF6_15 [Enterococcus phage iF6]	2.00E-29
131	vB_EfaH_163-131	110265	109843	140	16.51	4.48	hypothetical protein	hypothetical protein EfsSzw1_25 [Enterococcus phage EfsSzw-1]	1.00E-95
132	vB_EfaH_163-132	111659	110415	414	46.05	5.26	RNA ligase RtcB protein	protein RtcB [Enterococcus phage EfV12-phi1]	0.00E+00
133	vB_EfaH_163-133	111840	111652	62	7.47	5.67	hypothetical protein	hypothetical protein AVV19_gp210 [Enterococcus phage EFDG1]	5.00E-34
134	vB_EfaH_163-134	112672	111929	247	27.70	5	hypothetical protein	hypothetical protein HOU42_gp033 [Enterococcus phage EfV12-phi1]	0.00E+00
135	vB_EfaH_163-135	113253	112669	194	22.57	5.51	hypothetical protein	hypothetical protein PEf771_126 [Enterococcus phage PEf771]	1.00E-30
136	vB_EfaH_163-136	113353	113240	37	4.03	5.06	hypothetical protein	hypothetical protein HOR47_gp161 [Enterococcus phage EFP01]	4.00E-06
137	vB_EfaH_163-137	114204	113353	283	32.42	4.47	hypothetical protein	hypothetical protein [Enterococcus phage EFGrKN]	0.00E+00
138	vB_EfaH_163-138	114515	114201	104	12.59	5.12	hypothetical protein	hypothetical protein AVV19_p12 [Enterococcus phage EFDG1]	4.00E-68
139	vB_EfaH_163-139	115001	114519	160	17.82	5.56	hypothetical protein	hypothetical protein HOR47_gp156 [Enterococcus phage EFP01]	7.00E-105
140	vB_EfaH_163-140	115360	115130	76	9.02	4.71	hypothetical protein	hypothetical protein [Enterococcus phage vB_OCPT_Ben]	5.00E-45
141	vB_EfaH_163-141	115670	115374	98	11.43	5.4	hypothetical protein	hypothetical protein AVV19_gp196 [Enterococcus phage EFDG1]	2.00E-61
142	vB_EfaH_163-142	116116	115670	148	18.03	9.42	hypothetical protein	hypothetical protein HOR47_gp153 [Enterococcus phage EFP01]	1.00E-100
143	vB_EfaH_163-143	116696	116304	130	14.52	5.07	hypothetical protein	hypothetical protein HOR47_gp152 [Enterococcus phage EFP01]	4.00E-88

144	vB_EfaH_163-144	116952	116689	87	10.61	5.66	hypothetical protein	bacterial transferase hexapeptide repeat domain protein [Enterococcus phage EFP01]	1.00E-54
145	vB_EfaH_163-145	118662	117028	544	63.19	5.77	hypothetical protein	hypothetical protein HOR47_gp150 [Enterococcus phage EFP01]	0.00E+00
146	vB_EfaH_163-146	118998	118678	106	12.34	8.06	hypothetical protein	hypothetical protein HOR47_gp149 [Enterococcus phage EFP01]	9.00E-69
147	vB_EfaH_163-147	119812	119072	246	28.19	5.04	Serine/threonine phosphatase	serine/threonine protein phosphatase [Enterococcus phage iF6]	3.00E-176
148	vB_EfaH_163-148	120069	119812	85	10.84	9.45	hypothetical phage protein	hypothetical protein A2_49 [Enterococcus phage vB_EfaM_A2]	7.00E-53
149	vB_EfaH_163-149	120674	120066	202	23.67	6.46	Phosphoesterase	putative metallo-dependent phosphatase 1 [Enterococcus phage EFP01]	1.00E-145
150	vB_EfaH_163-150	121246	120671	191	22.25	5.06	hypothetical protein	phosphoesterase [Enterococcus phage PEf771]	2.00E-137
151	vB_EfaH_163-151	121777	121247	176	20.48	4.19	hypothetical protein	exonuclease [Enterococcus phage EFP01]	1.00E-125
152	vB_EfaH_163-152	122158	121808	116	13.32	6.14	hypothetical phage protein	hypothetical protein HOU42_gp054 [Enterococcus phage EfV12-phi1]	9.00E-74
153	vB_EfaH_163-153	122484	122155	109	12.93	5.92	hypothetical protein	hypothetical protein iF6_36 [Enterococcus phage iF6]	7.00E-46
154	vB_EfaH_163-154	122889	122554	111	13.10	5.09	hypothetical protein	hypothetical protein PEf771_105 [Enterococcus phage PEf771]	1.00E-46
155	vB_EfaH_163-155	123533	122970	187	20.99	5.01	hypothetical protein	hypothetical protein iF6_40 [Enterococcus phage iF6]	9.00E-133
156	vB_EfaH_163-156	123627	123535	30	3.30	5	hypothetical protein	hypothetical protein A2_62 [Enterococcus phage vB_EfaM_A2]	2.00E-24
157	vB_EfaH_163-157	123904	123701	67	7.88	4.77	hypothetical protein	hypothetical protein AVV19_p15 [Enterococcus phage EFDG1]	1.00E-38
158	vB_EfaH_163-158	124464	123904	186	21.63	9.16	hypothetical phage protein	hypothetical protein AVV19_p16 [Enterococcus phage EFDG1]	1.00E-136
159	vB_EfaH_163-159	125386	124517	289	31.59	7.67	hypothetical phage protein	hypothetical protein AVV19_gp169 [Enterococcus phage EFDG1]	0.00E+00
160	vB_EfaH_163-160	126098	125484	204	23.42	4.98	Ribonucleotide reductase of class III (anaerobic), activating protein (EC 1.97.1.4)	ribonucleotide reductase of class III (anaerobic), activating protein [Enterococcus phage EFDG1]	1.00E-148
161	vB_EfaH_163-161	126384	126091	97	10.98	5.51	hypothetical protein	hypothetical protein iF6_45 [Enterococcus phage iF6]	8.00E-63
162	vB_EfaH_163-162	127859	126384	491	56.39	6.69	Thymidylate synthase (EC 2.1.1.45)	thymidylate synthase [Enterococcus phage vB_EfaM_A2]	0.00E+00
163	vB_EfaH_163-163	128504	127875	209	24.25	10.32	hypothetical protein	hypothetical protein HOR47_gp132 [Enterococcus phage EFP01]	3.00E-147
164	vB_EfaH_163-164	129255	128506	249	29.55	4.61	hypothetical protein	hypothetical protein iF6_48 [Enterococcus phage iF6]	0.00E+00
165	vB_EfaH_163-165	129796	129329	155	17.26	4.53	hypothetical protein	nucleoside triphosphate pyrophosphorylase [Enterococcus phage iF6]	7.00E-111
166	vB_EfaH_163-166	130199	129888	103	12.09	6.84	hypothetical protein	hypothetical protein HOR47_gp129 [Enterococcus phage EFP01]	1.00E-66
167	vB_EfaH_163-167	130646	130284	120	14.10	6.41	hypothetical protein	HAD-like family phosphatase [Enterococcus phage iF6]	3.00E-85

168	vB_EfaH_163-168	130979	130740	79	8.94	8.58	Ribonucleotide reductase class Ib	putative ribonucleotide reductase [Enterococcus phage EFP01]	4.00E-50
169	vB_EfaH_163-169	131736	130972	254	28.46	9.3	Nicotinamide mononucleotide transporter	PnuC-like nicotinamide mononucleotide transporter [Enterococcus phage iF6]	0.00E+00
170	vB_EfaH_163-170	132569	131754	271	31.42	5.02	Deoxypurin kinase	deoxyguanosine kinase [Enterococcus phage PEf771]	
171	vB_EfaH_163-171	133637	132639	332	38.64	8.38	hypothetical protein	hypothetical protein [*Paenibacillus xylanexedens*]	4.00E-41
172	vB_EfaH_163-172	135866	133695	723	82.67	6.12	Ribonucleotide reductase of class III	ribonucleotide reductase of class III (anaerobic), large subunit [Enterococcus phage EFDG1]	0.00E+00
173	vB_EfaH_163-173	136286	136017	89	10.02	4.95	hypothetical phage protein	hypothetical protein EfsSzw1_70 [Enterococcus phage EfsSzw-1]	9.00E-57
174	vB_EfaH_163-174	137128	136298	276	31.56	9.74	hypothetical protein	hypothetical protein AVV19_gp147 [Enterococcus phage EFDG1]	0.00E+00
175	vB_EfaH_163-175	137452	137147	101	12.22	9.41	hypothetical protein	putative scaffolding protein [Enterococcus phage EFDG1]	1.00E-63
176	vB_EfaH_163-176	137768	137553	71	8.28	9.85	hypothetical phage protein	hypothetical protein AVV19_gp144 [Enterococcus phage EFDG1]	6.00E-44
177	vB_EfaH_163-177	139362	137827	511	57.73	6.63	hypothetical phage protein	hypothetical protein [Enterococcus phage vB_OCPT_Ben]	0.00E+00
178	vB_EfaH_163-178	140317	140093	74	8.42	9.95	hypothetical protein	hypothetical protein HOR47_gp117 [Enterococcus phage EFP01]	4.00E-45
		140696	140626				tRNA-Gly-TCC	tRNA-Gly-TCC	
		140996	140924				tRNA-Pro-TGG	tRNA-Pro-TGG	
		141383	141317				tRNA-Pseudo-ATG	tRNA-Pseudo-ATG	
		141513	141441				tRNA-Ala-TGC	tRNA-Ala-TGC	
		141594	141522				tRNA-Arg-TCT	tRNA-Arg-TCT	
179	vB_EfaH_163-179	141971	141603	122	14.38	5.02	hypothetical protein	hypothetical protein AVV19_gp140 [Enterococcus phage EFDG1]	2.00E-83
		142267	142181				tRNA-Ser-GGA	tRNA-Ser-GGA	
180	vB_EfaH_163-180	142545	142363	60	6.96	6.81	hypothetical protein	hypothetical protein [Enterococcus phage EFGrKN]	5.00E-36
		143377	143293				tRNA-Leu-TAG	tRNA-Leu-TAG	
		143626	143556				tRNA-His-GTG	tRNA-His-GTG	
181	vB_EfaH_163-181	143920	143699	73	8.38	3.92	hypothetical protein	hypothetical protein iF6_67 [Enterococcus phage iF6]	4.00E-44
182	vB_EfaH_163-182	144180	143929	83	10.60	9.79	hypothetical protein	hypothetical protein iF6_68 [Enterococcus phage iF6]	5.00E-54
		144408	144335				tRNA-Ile-GAT	tRNA-Ile-GAT	
		144633	144562				tRNA-Val-TAC	tRNA-Val-TAC	
		144938	144866				tRNA-Arg-ACG	tRNA-Arg-ACG	
		145114	145041				tRNA-Lys-TTT	tRNA-Lys-TTT	
		145322	145249				tRNA-Lys-CTT	tRNA-Lys-CTT	
		145586	145515				tRNA-Thr-TGT	tRNA-Thr-TGT	

183	vB_EfaH_163-183	146703	145612	363	41.75	4.96	hypothetical protein	hypothetical protein AVV19_p36 [Enterococcus phage EFDG1]	0.00E+00
		146816	146743				tRNA-Phe-GAA	tRNA-Phe-GAA	
		147010	146939				tRNA-Cys-GCA	tRNA-Cys-GCA	
		147094	147023				tRNA-Trp-CCA	tRNA-Trp-CCA	
		147383	147311				tRNA-Met-CAT	tRNA-Met-CAT	
		148956	148868				tRNA-Ser-GCT	tRNA-Ser-GCT	
		149137	149066				tRNA-Gln-TTG	tRNA-Gln-TTG	
		149299	149229				tRNA-Asn-GTT	tRNA-Asn-GTT	
184	vB_EfaH_163-184	150073	149735	112	12.76	5.68	hypothetical protein	hypothetical protein iF6_69 [Enterococcus phage iF6]	2.00E-75
185	vB_EfaH_163-185	150475	150128	115	13.33	4.91	hypothetical phage protein	hypothetical protein HOR47_gp107 [Enterococcus phage EFP01]	1.00E-76
186	vB_EfaH_163-186	150772	150515	85	9.67	9.3	hypothetical phage protein	hypothetical protein AVV19_gp132 [Enterococcus phage EFDG1]	8.00E-51
